# Changes in Resting-State Spontaneous Brain Activity in Patients With Allergic Rhinitis: A Pilot Neuroimaging Study

**DOI:** 10.3389/fnins.2021.697299

**Published:** 2021-07-14

**Authors:** Ziang Gao, Xixiang Chen, Rong Xiang, Wei Zhang, Lu Tan, Wenjun Fan, Peiqiang Liu, Hao Lv, Yu Xu

**Affiliations:** ^1^Department of Otolaryngology-Head and Neck Surgery, Renmin Hospital of Wuhan University, Wuhan, China; ^2^Department of Radiology, Renmin Hospital of Wuhan University, Wuhan, China

**Keywords:** neuroimaging, allergic rhinitis, resting-state fMRI, precuneus, anterior cingulate cortex, cognition, memory

## Abstract

**Background:**

Allergic rhinitis (AR) is an inflammatory disorder of the nose caused by immunoglobulin E (IgE)-mediated immune response to allergens. Apart from the typical symptoms of sneezing, itching, rhinorrhea, and nasal congestion, behavioral complications were also reported to be associated with the progression of AR, such as cognitive deficits, mood changes, memory decline, attention deficiency, poor school performance, anxiety, and depression. Recent human studies have suggested that alterations in brain function caused by allergen exposure may precipitate high levels of anxiety and emotional reactivity in asthma patients. But until now, there is no direct evidence of the relationship between brain activity and allergic rhinitis.

**Methods:**

Resting-state functional magnetic resonance imaging (rs-fMRI) was used to excavate whether there remain functional changes of brain activity in AR patients. We measured the amplitude of low-frequency fluctuation (ALFF) and the *z* conversion of ALFF (zALFF) in 20 patients with AR and 20 age- and sex-matched healthy controls (HCs) using the rs-fMRI data.

**Results:**

Compared with healthy controls, AR patients exhibited lower ALFF values in the precuneus (PCUN) and higher ALFF values in the anterior cingulate cortex (ACC). The ALFF values of these features were significantly correlated with the visual analog scale (VAS) scores, the Rhinoconjunctivitis Quality of Life Questionnaire (RQLQ) scores, the subscales of RQLQ, and specific IgE, partly.

**Conclusion:**

We found changes in resting-state spontaneous brain activity in AR patients with hypoactivity in the PCUN and hyperactivity of the ACC. The brain-related symptoms of AR might be another potential clinical intervention target for improving the life quality of AR patients. Further attention to brain activity is essential for a deeper understanding of AR.

## Introduction

Allergic rhinitis (AR) is an inflammatory disorder of the nasal mucosa induced by immunoglobulin E (IgE)-mediated immune response to allergens (Cheng L. et al., 2018; Meng et al., 2019). In the past decades, AR has become a worldwide public health problem and affected up to 40% of the population. Although not life-threatening, the symptoms of AR as well as the comorbid diseases are frequently bothersome, adversely affecting work, psychological health, and quality of life, imposing a significant socioeconomic burden on both the individual and society.

The characteristic symptoms of AR are sneezing, itching, rhinorrhea, and nasal congestion (Brożek et al., 2017; Cheng L. et al., 2018; Meng et al., 2019). Apart from these typical symptoms, behavioral problems were also reported in AR patients, such as impaired quality of life (QOL), disturbed sleep, and unsatisfactory performance in school and at work (Bousquet et al., 2012; Brożek et al., 2017; Cheng L. et al., 2018; Meng et al., 2019), as well as brain-related symptoms including psychological problems, memory problems, cognition problems, concentration deficits, and mood changes (Ozdoganoglu et al., 2012; Trikojat et al., 2017). How these brain-related symptoms occur is unclear. Recent human studies have suggested that alterations in brain function could be caused by allergen exposure and might precipitate high levels of anxiety and emotional reactivity in asthma patients (Damoiseaux et al., 2012; Cui et al., 2020). Callebaut et al. (2020) found the activation of different brain regions upon nasal histamine provocation in AR patients. With an AR rat model, Yang et al. (2018) found inflammatory responses in the hippocampus region. In our previous study, neuroinflammation was also observed in the olfactory bulb in an allergic rhinitis mouse model (Lv et al., 2021). These studies suggested that there might be aberrant brain activity in the brain regions of AR patients that is responsible for the brain-related symptoms. But until now, there is no direct evidence of the relationship between brain activity and allergic rhinitis.

Neuroimaging techniques could help detect structural and functional brain abnormalities at an early stage. Studies incorporating structural and functional magnetic resonance imaging (MRI) can provide more comprehensive information on the underlying mechanisms of the various pathways in the pathogenesis of diseases (Dodd et al., 2012). Resting-state functional MRI (rs-fMRI) is an excellent tool for probing neural networks and has been widely used to investigate changes in the global functional network connectivity and local spontaneous neuronal activity in the brain at rest. Contrastingly, in the subjective cognitive decline (SCD) disease model, some studies have failed to find structural changes, but have reported differences in brain function, as measured by blood oxygen level-dependent functional MRI (BOLD fMRI) (Scarapicchia et al., 2019). Notably, some neuroimaging findings in Alzheimer’s disease (AD) suggest that changes in brain function may actually precede changes in brain structure (Damoiseaux et al., 2012). The amplitude of low-frequency fluctuation (ALFF) is an rs-fMRI method that may serve as a surrogate for neural activity at a single-voxel level (Dodd et al., 2012; Cui et al., 2014, 2020; Yang et al., 2018; Scarapicchia et al., 2019; Callebaut et al., 2020; Lv et al., 2021). Given that, an ALFF analysis may provide important information on the spontaneous brain activity pattern specific to AR and on the difference between AR patients and healthy controls (HCs).

In this study, our primary goal was to observe the changes in resting-state spontaneous brain activity in ALFF in AR patients, aiming to make an initial study of the mechanisms of the brain-related symptoms in AR. Taking into account that few studies to date have examined the relationship between AR and brain-related disorders in individuals with AR, and that none of the fMRI measures of BOLD variability in this group was reported, we also investigated the relationship between alterations in ALFF and clinical indexes, as well as allergy indicators, using correlation analysis for our pilot neuroimaging study.

## Materials and Methods

### Participants

A total of 20 AR patients were recruited from the Otorhinolaryngology Department of Renmin Hospital of Wuhan University. The inclusion criteria were as follows: 18–50 years old and moderate to severe AR for more than 1 year. The diagnostic criteria for AR are according to the Allergic Rhinitis and its Impact on Asthma Guidelines (ARIA) (Brożek et al., 2017): (1) positive skin prick tests or circulating levels of allergen-specific IgE antibody ≥ 0.7 kU/L and (2) clinical history or identified allergen. The exclusion criteria were allergic asthma; moderate to severe atopic dermatitis; any autoimmune disorder; specific immunotherapy during the past 3 years; any severe chronic inflammatory disease; any neuropsychiatric disease; any history of brain surgery, alcohol, or drug abuse; contraindications to MR examinations; and pregnancy or breastfeeding. Demographic and clinical data such as age, gender, year of education, disease duration, visual analog scale (VAS) score, and the Rhinoconjunctivitis Quality of Life Questionnaire (RQLQ) score of each patient were collected.

Twenty age-, sex-, and education level-matched HCs were also enrolled as a control group in the present study. All of the subjects were right-handed according to the Edinburgh Handedness Inventory.

This study was approved by the Human Research Ethics Committee of Renmin Hospital of Wuhan University (Wuhan, China). Written informed consent was given to all the participants (approval WDRY2020-K233).

### MRI Acquisition Data Preprocessing

Scanning of this study was performed at the Radiology Department of Renmin Hospital of Wuhan University (Wuhan, China) using a 3-T MR scanner (Discovery MR 750 W System; GE Healthcare, Milwaukee, WI, United States) with an eight-channel head coil. Structural T1-weighted images were acquired with the following parameters: 192 slices, repetition time/echo time = 8.5/3.3, thickness = 1.00 mm, no intersection gap, acquisition matrix = 256 × 256, field of view = 240 × 240 mm^2^, and flip angle = 12°. Echo planar images (EPIs) were acquired with the following parameters: 40 slices, repetition time = 2,000 ms, echo time = 25 ms, thickness = 3.0 mm, gap = 1.2 mm, acquisition matrix = 64 × 64, flip angle = 90°, field of view = 240 × 240 mm^2^, and voxel size = 3.6 × 3.6 × 3.6 mm^3^ during an 8-min scanning time. Three EPI series were collected from all participants, and they underwent MRI scans with their eyes closed, but not sleeping.

### Functional MRI Data Acquisition and Preprocessing

Resting-state functional images were preprocessed through Statistical Parametric Mapping SPM12^1^ and the toolbox for Data Processing Assistant and Analysis for Brain Imaging^2^ software.

Steps could be briefly generalized as follows: (a) Remove the first 10 time point functional images to achieve equilibrium because of lack of adaptation to the scanning environment and unstable initial MRI signal. (b) The intra-volume temporal mismatch and intervolume spatial displacement were subsequently corrected. EPIs were normalized to the Montreal Neurological Institute (MNI) standard space and resampled to a resolution of 3 × 3 × 3 mm^3^. Normalized images were smoothed spatially with a 6 × 6 × 6-mm^3^ full width at a half maximum Gaussian kernel. Denoising methods were applied, including bandpass filtering and nuisance covariate regression (linear trend, Friston 24-parameter head motion parameters, white matter signal, cerebrospinal fluid, and global signal). Subjects with head motion that exceeded the maximum displacement of 2 mm at each axis and an angular motion of 2° for each axis (*x*, *y*, *z*, pitch, roll, and yaw) were excluded from further analysis. Using this criterion, seven AR subjects and three control subjects were excluded, which resulted in 20 AR and 20 control subjects.

### Amplitude of Low-Frequency Fluctuation

The BOLD time series for each voxel was first converted to the frequency domain using fast Fourier transform. The square root of the power spectrum was subsequently computed and averaged across the specified frequency range (0.01--0.08 Hz) at each voxel. The averaged square root was considered the ALFF. Finally, this value was transformed using Fisher’s *z* transformation amplitude of low-frequency fluctuations (zALFF) and used for subsequent group-level analysis. Calculations were performed using REST software version 1.8^3^. Then, the mean ALFF value was extracted by averaging the ALFF values over all voxels for each individual.

### Statistical Analysis

Demographic and clinical data such as age, gender, year of education, disease duration, VAS score, and RQLQ score were analyzed using the Statistical Package for the Social Sciences version 26.0 (IBM Corporation, Armonk, NY, United States) between the two groups. The two-tailed *t*-test was performed for variables and the statistical threshold set at *p* < 0.05.

The two-sample *t*-test was used to compare the zALFF values in each voxel of the two groups (two-tailed, voxel-level: *p* < 0.01; Gaussian random field theory correction, cluster-level: *p* < 0.05). Pearson’s correlation analysis was used to investigate the relationship between the mean ALFF values in different brain regions and clinical performance for AR patients. Spearman’s correlation analysis was employed to investigate the correlation between the ALFF *z*-values in the precuneus (PCUN) and specific IgE. Partial correlation analyses were performed in the comparisons above, excluding the effects of gender, age, and years of education. A *p*-value < 0.05 was considered to represent a significant difference.

## Results

### Demographic and Clinical Data

There were no significant differences in age or sex between the HC and AR groups (*p* > 0.05; see Table 1). Table 1 provides the demographics of the samples and the VAS and RQLQ scores.

**TABLE 1:** Demographic and clinical characteristics between allergic rhinitis (AR) patients and the healthy control (HC) group.

**Characteristics**	**HC (*n* = 20)**	**AR (*n* = 20)**	***p*-value**
Age (years)	32.3 ± 9.1	37.1 ± 8.3	0.104
Male/female (*n*)	9/11	9/11	–
Education (years)	15.2 ± 1.6	14.2 ± 3.7	0.291
VAS scores	–	46.6 ± 12.5	–
Overall	–	7.9 ± 2.2	–
Sneezing	–	7.3 ± 2.3	–
Rhinorrhea	–	7.2 ± 2.9	–
Itching	–	6.1 ± 2.9	–
Congestion	–	7.7 ± 2.7	–
Eye itching	–	6.0 ± 1.6	–
Lacrimation	–	4.8 ± 2.3	–
Impact on life	–	7.6 ± 2.3	–
RQLQ scores	–	95.5 ± 28.9	–
Activity limitation	–	10.3 ± 3.9	–
Reading	–	3.2 ± 1.8	–
Practice	–	2.9 ± 1.7	–
Social activities	–	4.2 ± 1.7	–
Sleep disturbance	–	9.9 ± 5.8	–
Difficulty getting to sleep	–	3.4 ± 2.1	–
Wake up during the night	–	2.9 ± 2.2	–
Restless	–	3.7 ± 2.2	–
Non-nasal/eye symptoms	–	22.6 ± 10.0	–
Fatigue	–	3.7 ± 2.0	–
Thirsty	–	3.0 ± 2.2	–
Productivity degradation	–	2.9 ± 1.9	–
Tired	–	3.4 ± 2.0	–
Attention deficit	–	3.5 ± 1.4	–
Headache	–	2.9 ± 1.8	–
Exhausted	–	3.4 ± 2.0	–
Practical problems	–	13.4 ± 5.4	–
Have to carry tissues	–	4.4 ± 1.9	–
Need to rub nose/eyes	–	4.5 ± 1.9	–
Need to blow nose	–	4.5 ± 1.8	–
Nasal symptoms	–	16.4 ± 5.1	–
Congestion	–	4.7 ± 1.7	–
Rhinorrhea	–	4.5 ± 1.7	–
Sneezing	–	4.6 ± 1.5	–
Postnasal drip	–	2.7 ± 2.1	–
Eye symptoms	–	10.3 ± 5.3	–
Eye hyperemia	–	2.2 ± 2.0	–
Lacrimation	–	2.9 ± 1.8	–
Eye ache	–	1.7 ± 1.4	–
Eye itching	–	3.6 ± 1.5	–
Emotional function	–	11.2 ± 4.8	–
Depression	–	1.5 ± 1.5	–
Impatient or restless	–	3.2 ± 1.7	–
Irritable	–	2.7 ± 2.2	–
Embarrassed	–	3.9 ± 1.8	–

*VAS, visual analog scale; RQLQ, Rhinoconjunctivitis Quality of Life Questionnaire.*

### ALFF Alterations Between AR Patients and HCs

**FIGURE 1:**
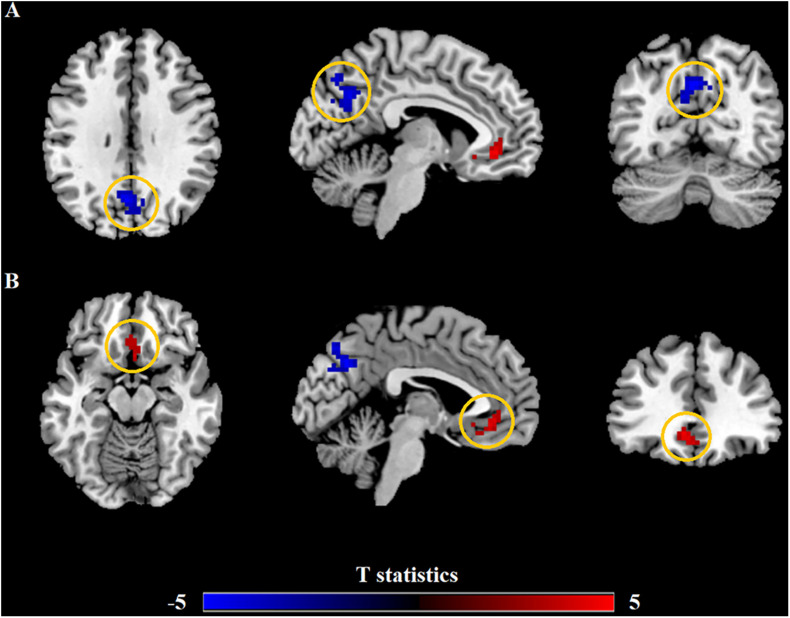
Differences in the amplitude of low-frequency fluctuation (ALFF) between allergic rhinitis (AR) patients and the healthy control (HC) group. **(A)** The AR group showed significant lower ALFF values in the left precuneus (PCUN, in *blue*) compared with the HC group. *Clusters in blue* also included part of the right PCUN (16 voxels) and bilateral cuneus (25 voxels). **(B)** The AR group showed significant higher ALFF values in the left anterior cingulate cortex (ACC, in *red*) compared with the HC group. *Clusters in red* also included part of the bilateral medial orbital superior frontal gyrus (16 voxels), bilateral rectus (16 voxels), and bilateral olfactory (8 voxels). *p* < 0.01, cluster level; *p* < 0.05, family-wise error (FWE) correction.

**FIGURE 2:**
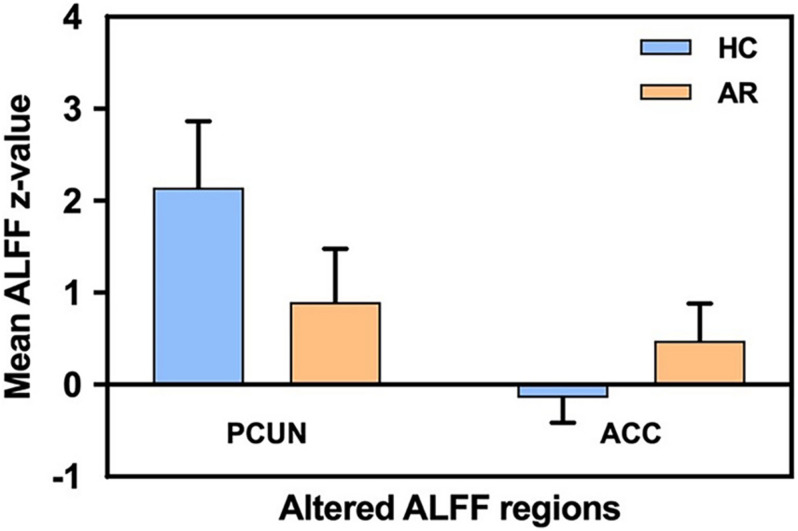
Mean ALFF signal values for the altered regional brain areas between AR patients and HCs. ALFF, amplitude of low-frequency fluctuation; AR, allergic rhinitis; HCs, healthy controls; PCUN, precuneus; ACC, anterior cingulate cortex.

Relevant information of the mean ALFF values is shown in Table 2 and the visual images shown in Figure 1. Our findings revealed that AR patients showed significantly lower ALFF values in the PCUN and significantly higher ALFF values in the anterior cingulate cortex (ACC) compared to the HCs. The average ALFF *z*-values of the altered brain regions are shown in Figure 2, and the difference was statistically significant.

**TABLE 2:** Two-sample *t*-test differences between AR patients and HCs using the ALFF method.

**Brain region**	**R/L**	**No. of voxels**	**MNI (*x*, *y*, *z*)**			**Peak**
AR < HC						
PCUN	L	105	0	–66	36	–5.1729
AR > HC						
ACC	L	61	–6	39	–6	4.7576

*AR, allergic rhinitis; HCs, healthy controls; ALFF, amplitude of low-frequency fluctuation; FWE, family-wise error; R/L, right/left hemi-cerebrum; MNI, Montreal Neurological Institute; PCUN, precuneus; ACC, anterior cingulate cortex.*

*p < 0.01, cluster level; p < 0.05, FWE correction.*

### Correlation Results

**FIGURE 3:**
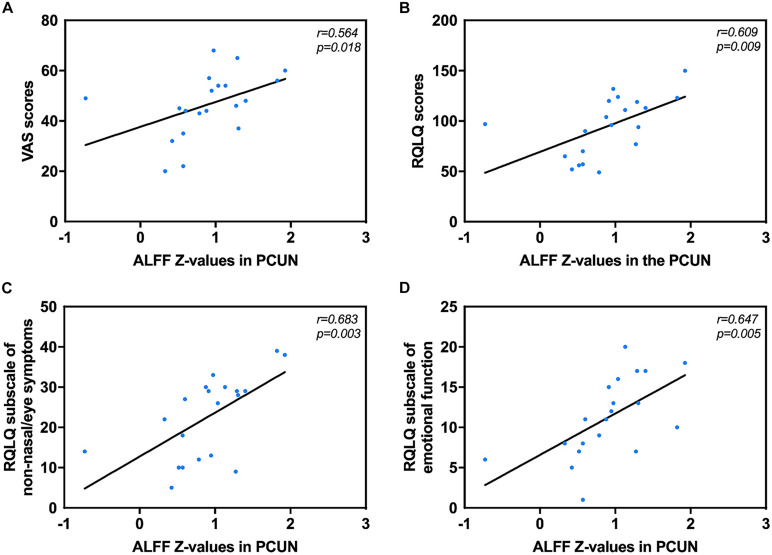
Significant correlation analyses between AR patients’ ALFF values in the PCUN and clinical indexes. **(A)** PCUN and VAS scores: *n* = 20, *r* = 0.564, *p* = 0.018. **(B)** PCUN and RQLQ scores: *n* = 20, *r* = 0.609, *p* = 0.009. **(C)** PCUN and subscale of non-nasal/eye symptoms of RQLQ scores: *n* = 20, *r* = 0.683, *p* = 0.003. **(D)** PCUN and subscale of emotion symptoms of RQLQ scores: *n* = 20, *r* = 0.647, *p* = 0.005. ALFF, amplitude of low-frequency fluctuation; AR, allergic rhinitis; PCUN, precuneus; VAS, visual analog scale; RQLQ, Rhinoconjunctivitis Quality of Life Questionnaire.

Correlation analysis revealed that the mean ALFF values in the PCUN displayed significant positive correlations with the VAS (*r* = 0.562, *p* = 0.019) and RQLQ (*r* = 0.623, *p* = 0.008) scores. Moreover, the ALFF values in the PCUN also had significant positive correlations with the RQLQ subscale of non-nasal/eye symptoms (*r* = 0.683, *p* = 0.003) and emotional function (*r* = 0.647, *p* = 0.005), as shown in Figure 3. There was no significant correlation between the ALFF values in the PCUN and other subscales (*p* > 0.05).

**FIGURE 4:**
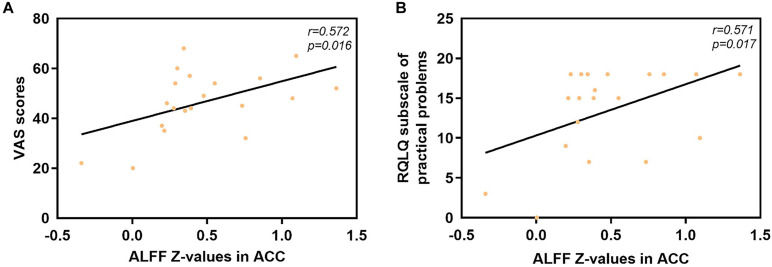
Significant correlation analyses between AR patients’ ALFF values in the ACC and clinical indexes. **(A)** ACC and VAS scores: *n* = 20, *r* = 0.572, *p* = 0.016. **(B)** ACC and subscale of practical problems of RQLQ scores: *n* = 20, *r* = 0.571, *p* = 0.017. ALFF, amplitude of low-frequency fluctuation; AR, allergic rhinitis; ACC, anterior cingulate cortex; VAS, visual analog scale; RQLQ, Rhinoconjunctivitis Quality of Life Questionnaire.

The mean ALFF values in the ACC displayed significant positive correlations with the VAS score (*r* = 0.572, *p* = 0.016) and the RQLQ subscale of practical problems (*r* = 0.571, *p* = 0.017), as shown in Figure 4. However, no significant correlation was observed between the mean ALFF values in the ACC and other subscales of the RQLQ score (*p* > 0.05).

**FIGURE 5:**
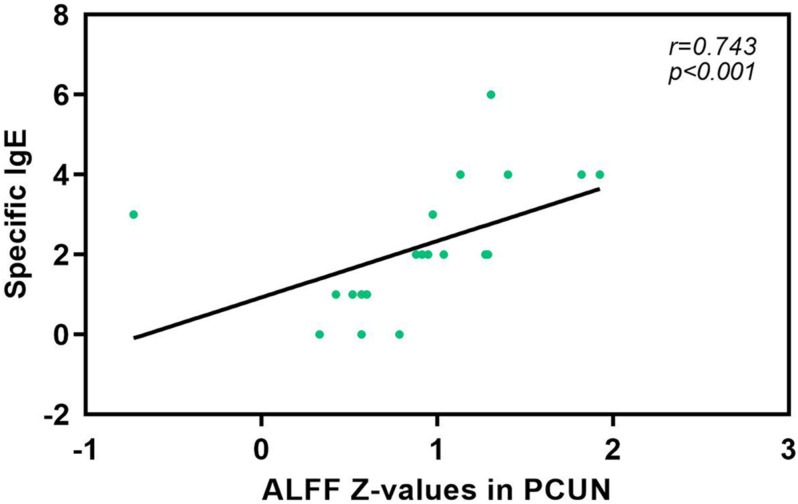
Significant correlation analysis between AR patients’ ALFF values in the PCUN and specific IgE results. Spearman’s correlation analysis between the ALFF *z*-values in the PCUN and specific IgE: *n* = 20, *r* = 0.743, *p* < 0.001. ALFF, amplitude of low-frequency fluctuation; AR, allergic rhinitis; PCUN, precuneus.

**FIGURE 6:**
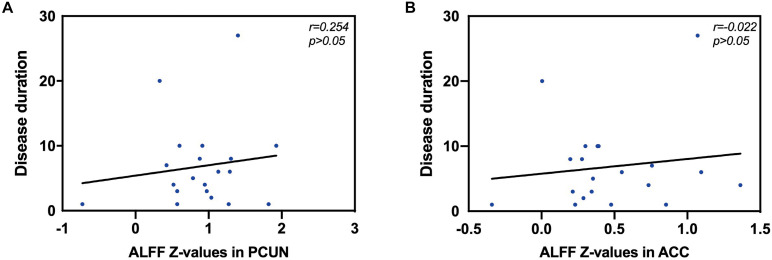
Correlation analysis between disease duration (years) and the ALFF values in the PCUN and ACC. **(A)** Correlation analysis between disease duration and the ALFF values in the PCUN: *n* = 20, *r* = 0.254, *p* > 0.05. **(B)** Correlation analysis between disease duration and the ALFF values in the ACC: *n* = 20, *r* = 0.022, *p* > 0.05. ALFF, amplitude of low-frequency fluctuation; PCUN, precuneus; ACC, anterior cingulate cortex.

We also analyzed the fMRI results and the indexes of allergy. We found significant positive correlations between AR patients’ ALFF in the PCUN and the specific IgE results (*r* = 0.743, *p* < 0.001), as shown in Figure 5. However, the correlation analysis between disease duration and the ALFF values in the PCUN and ACC showed insignificant results (PCUN: *r* = 0.254, *p* > 0.05; ACC: *r* = 0.022, *p* > 0.05), as shown in Figure 6. Additionally, we also performed a multivariate linear regression analysis on ALFF *z*-values in the PCUN (*F* = 41.150, *R*^2^ = 0.864) and ACC (*F* = 24.383, *R*^2^ = 0.787), as shown in Table 3.

**TABLE 3:** Estimation results of the multivariate linear regression coefficients on the ALFF *z*-values in the PCUN and ACC.

**Factor**	***B***	**Standard error**	***t***	***p***	**VIF**
Constant	–0.418	0.172	–2.424	0.028	–
VAS score	0.011	0.005	2.148	0.047	2.287
RQLQ score	0.006	0.002	2.526	0.022	3.149
Specific IgE	0.118	0.037	3.231	0.005	2.134
*The dependent variable for the above data is the ALFF *z*-value in the PCUN (*F* = 41.150, *R*^2^ = 0.864)					
Constant	–0.855	0.167	–5.123	0.000	–
VAS score	0.018	0.005	3.880	0.001	1.903
RQLQ score	0.004	0.002	2.123	0.050	2.045
Specific IgE	0.039	0.029	1.321	>0.05	1.304
*The dependent variable for the above data is the ALFF z-value in the ACC (*F* = 24.383, *R*^2^ = 0.787).					

*VIF, variance inflation factor; VAS, visual analog scale; RQLQ, Rhinoconjunctivitis Quality of Life Questionnaire; IgE, immunoglobulin E*.

## Discussion

**FIGURE 7:**
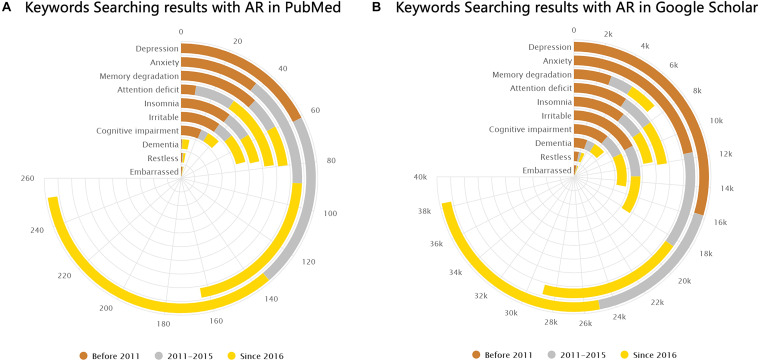
Keyword search results in PubMed and Google Scholar. **(A,B)** Number of results from PubMed and Google Scholar (by February 2021) shown separately for the following keywords: “(allergic rhinitis) AND (depression),” “(allergic rhinitis) AND (anxiety),” “(allergic rhinitis) AND (memory degradation),” “(allergic rhinitis) AND (attention deficit),” “(allergic rhinitis) AND (insomnia),” “(allergic rhinitis) AND (irritable),” “(allergic rhinitis) AND (cognitive impairment),” “(allergic rhinitis) AND (dementia),” “(allergic rhinitis) AND (restless),” and “(allergic rhinitis) AND (embarrassed).”

Brain-related symptoms are an important part of AR. By the statistics of the keyword search results in PubMed and Google Scholar databases, we found that several brain-related symptoms in AR have been mentioned frequently, especially in recent years, as shown in Figure 7. Epidemiologic studies showed that a diagnosis of major depressive disorder is 1.7 times higher in AR patients compared to that in non-allergic subjects (Cuffel et al., 1999; Hurwitz and Morgenstern, 1999). Despite anxiety and depression, disorders relating to cognition, attention, and memory in AR are also common clinically. A cross-sectional study found that allergic rhinitis was associated with loss of energy and concentration difficulty (Robles-Figueroa et al., 2020). Kremer et al. illustrated that AR was related to a significantly impaired psychological wellbeing and perceived impaired cognitive functioning (Kremer et al., 2002). Pollen-allergic AR patients delivered an increased amount of total errors in specific measurements of spatial working memory during the pollen season compared to those in the control group (Papapostolou et al., 2020). It has been shown that untreated allergic patients experience a subtle slowed speed of cognitive processing (Marshall et al., 2000). Moreover, recent studies have proposed that pediatric allergic airway disease was associated with declines in cognitive function and school attendance (Yamasaki et al., 2020). As for attention deficits, AR is also reported to be closely related to attention-deficit hyperactivity disorder (ADHD) in previous studies (Brawley et al., 2004; Melamed and Heffron, 2016; Yang et al., 2016; Feng et al., 2017; Miyazaki et al., 2017; Wang et al., 2018; Chen et al., 2019; Guo et al., 2020). Children with ADHD were found to be more likely to have AR than are their counterparts (Miyazaki et al., 2017). Brain-related symptoms like cognitive impairment (CI), mood changes, and attention deficits also exist in other allergic diseases such as asthma and atopic dermatitis. It is worth mentioning that CI was largely observed in adults with asthma (Rhyou and Nam, 2020). Additionally, patients with vasomotor rhinitis also suffer from psychological damage (Zhang et al., 2020).

However, most of these studies are based on one-item self-reports regarding the absence or presence of brain-related symptoms and, therefore, lack a subjective and visualized assessment of psychological impairments in AR patients. The underlying pathophysiological mechanisms of clinically relevant psychological disorders in AR patients remain elusive as well. rs-fMRI might provide a tool for visualizing the changes of the different brain regions in AR.

**FIGURE 8:**
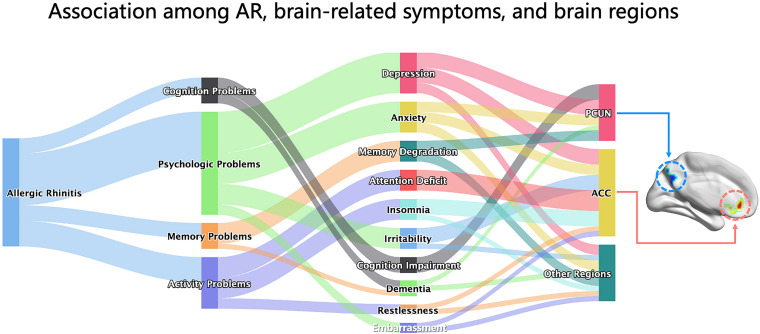
Associations among AR, brain-related symptoms, and brain regions. The Sankey diagram showed the associations among AR, brain-related symptoms (including depression, anxiety, memory degradation, attention deficit, insomnia, irritability, cognitive impairment, dementia, restlessness, and embarrassment, which were grouped into four categories for cognition problems, psychological problems, memory problems, and activity problems), and brain regions (PCUN, ACC, and others) abstractly based on the relationship between them from knowledge of AR literatures. The brain map *on the right* was our result of the ALFF analysis presented by the BrainNet toolbox in SPM. AR, allergic rhinitis; PCUN, precuneus; ACC, anterior cingulate cortex; ALFF, amplitude of low-frequency fluctuation.

In this study, we report on the changes in resting-state spontaneous brain activity in AR patients. ALFF analysis was used to investigate alterations of the BOLD signal and the correlation between brain areas and clinical data. We found that AR patients mainly exhibited a significant lower ALFF in the PCUN and a significant higher ALFF in the ACC. Moreover, the ALFF values showed significant correlations with clinical indexes, and the ALFF in the PCUN reflected positive correlations with the specific IgE results of AR patients. Patients with AR may experience changes in brain function, and these changes may result in CI, memory degradation, anxiety–mood disorder, and attention deficits. The connections between the brain-related symptoms and brain function regions are shown abstractly in Figure 8 in the form of a Sankey diagram and related to our results in the brain map. To our knowledge, this is the first report on the resting-state spontaneous brain activity in AR patients showing the activation of different brain regions and providing important information about the brain circuitry changes of AR patients.

The PCUN stands out for its distinctive role in fundamental cognitive functioning (Cavanna and Trimble, 2006). Studies have revealed that PCUN activation increased during memory retrieval (Fletcher et al., 1995; Maddock et al., 2001; Lundstrom et al., 2005; Hebscher et al., 2020), emotion processing (Fletcher et al., 1995; Maddock et al., 2001, 2003; Lundstrom et al., 2005; Cavanna and Trimble, 2006; Hebscher et al., 2020), and reward monitoring (Hayden et al., 2008). It was demonstrated that the PCUN plays an important role in memory and novelty detection (Lundstrom et al., 2003; Gur et al., 2007; Kafkas and Montaldi, 2014; Gilmore et al., 2015), especially during episodic (Fletcher et al., 1995; Maddock et al., 2001; Lundstrom et al., 2005) and autobiographical (Addis et al., 2004; Eustache et al., 2004) memory tasks within the regions of the default mode network (DMN) (Li et al., 2019). The DMN appears to play a commanding role in the large-scale functional organization in the resting state without tasks (Raichle, 2015), which is closely related to brain functions such as monitoring the internal and external environments, maintaining consciousness awakening, emotional processing, self-introspection, and extraction of episodic memory (Andrews-Hanna, 2012). The PCUN is also one of the brain areas associated with anxiety (Lai, 2018), sleep, and depressive problems, together with the orbitofrontal cortex (OFC) (Cheng W. et al., 2018). Consistently, the OFC is related to the olfactory system and has increased functional connectivity with the PCUN, as shown in an fMRI study (Cheng et al., 2016). Olfactory function was correlated with OFC in Alzheimer’s dementia and Parkinson’s disease dementia (Lee et al., 2020), which indicates that the PCUN closely relates to the olfactory system and neurodegeneration-related functions.

The ACC has a near-ubiquitous presence in the neuroscience of cognition within the region of the limbic system (Shenhav et al., 2013; Rolls, 2019). It has been implicated in a diversity of functions, from reward processing and performance monitoring to the execution of control and action selection (Shenhav et al., 2013). The ACC also receives information from the OFC about reward and non-reward outcomes. It is involved in emotion for it connects rewards to actions (Rolls, 2019). The ACC is a critical hub for mood disorders (Barthas et al., 2015) and is involved in the appraisal and expression of negative emotion, especially depression, anxiety, and fear (Etkin et al., 2011; Godlewska et al., 2018; Rolls et al., 2019). Irritability is a common clinical problem in AR patients. The ACC was found to present hyperactivity in irritable youth (Leibenluft, 2017). Attention deficit was also found to have a relationship with ACC, as mentioned above in ADHD (Bauer et al., 2018; Naaijen et al., 2018; Vogt, 2019), which was consistent with our clinical findings of concentration disorders in AR patients. Fatigue (Capuron et al., 2005) and embarrassment (Sturm et al., 2013; Morita et al., 2014) were reported to have a connection with ACC as well. Besides, a pharmacological MRI design was undertaken in AR patients allergic to house dust mite, and the results showed that several brain regions, including the ACC, were activated after nasal histamine provocation (Callebaut et al., 2020). However, rs-fMRI is more reflective of a patient’s usual functional brain activity than is task-state functional MRI.

Our neuroimaging results also corresponded with our clinical findings. In this study, we showed significant correlations between the ALFF values of AR patients and clinical indexes. We found that the VAS scores, RQLQ scores, and the RQLQ subscales of “non-nasal/eye symptoms and emotional function” had positive correlations with the ALFF in the PCUN. As shown in Table 2, non-nasal/eye symptoms include indexes of “fatigue, thirsty, productivity degradation, tired, attention deficit, headache, and exhausted” and emotional function includes indexes of “depression, impatient or restless, irritable, and embarrassed.” In the ACC, we found that the VAS scores and the RQLQ subscale of practical problems including indexes of “have to carry tissues, need to rub nose/eyes, and need to blow nose” had positive correlations. Moreover, we found that the specific IgE index had positive correlations with the ALFF values, which indicated the potential relationship between allergic immunological abnormalities and the brain-related symptoms of AR patients. In addition, we also found statistically significant effects of the VAS scores, RQLQ scores, and specific IgE on the ALFF values in the PCUN and ACC through multivariate linear regression analysis, as shown in Table 3. These associations suggest that more severe clinical symptoms would indicate stronger functional brain activity.

Given the evidence for the relationship between allergic rhinitis and brain-related symptoms, the possible pathogenesis might include, but is not limited to, the following perspectives: (1) physiological effects—the physiological role of nasal obstruction and its impairing impact on sleep may, together, subsequently affect psychiatric symptoms negatively (Léger et al., 2006; Fang et al., 2010); (2) cytokines—pro-inflammatory cytokines could access the central nervous system and interact with a cytokine network in the brain, which may virtually influence every aspect of brain-related behavior through different pathways (Capuron and Miller, 2011); (3) neuroinflammation—the involvement of the microglia and astrocytes in the initiation of both pro- and anti-inflammatory events indirectly points toward the degeneration of neurons (De Virgilio et al., 2016; Gelders et al., 2018; Passamonti et al., 2019); and (4) genetics—there exists a possible shared genetic risk between allergic disorders and depression (Wamboldt et al., 2000).

Our study also has limitations and caveats. The number of our case samples was small, which limited our comprehensive understanding of the relationship between the activated brain regions and the severity of AR. Moreover, we did not conduct a cognitive or psychological analysis using professional scales or a questionnaire for each patient. We will continue to collect clinical samples for further research. To the best of our knowledge, our current study is one of the first resting-state functional neuroimaging studies in the field of allergic rhinitis and is one of the first studies to connect clinical indexes with fMRI values. According to our results, early intervention and therapy for brain-related symptoms in AR will be recommended. AR patients, otherwise, may have a risk of AD or other neurodegeneration diseases.

## Data Availability Statement

The raw data supporting the conclusions of this article will be made available by the authors, without undue reservation.

## Ethics Statement

The studies involving human participants were reviewed and approved by the Human Research Ethics Committee of Renmin Hospital of Wuhan University (Wuhan, China). The patients/participants provided their written informed consent to participate in this study.

## Author Contributions

ZG and YX designed the whole study. RX, WZ, and LT selected the patients, analyzed the data, and prepared the questionnaires. XC and ZG performed the scanning for participants. PL undertook the statistical analysis. WF and HL participated in the interpretation of data. ZG and HL wrote the manuscript. PL and YX revised the manuscript. All authors read and approved the final manuscript.

## Conflict of Interest

The authors declare that the research was conducted in the absence of any commercial or financial relationships that could be construed as a potential conflict of interest.

## Footnotes

1 http://www.fil.ion.ucl.ac.uk/spm/

2 http://rfmri.org/dpabi

3 www.restfmri.net

## References

[B1] Addis D. R. McIntosh A. R. Moscovitch M. Crawley A. P. McAndrews M. P. (2004). Characterizing spatial and temporal features of autobiographical memory retrieval networks: a partial least squares approach. *Neuroimage* 23 1460–1471. 10.1016/j.neuroimage.2004.08.007 15589110

[B2] Andrews-Hanna J. R. (2012). The brain’s default network and its adaptive role in internal mentation. *Neuroscientist* 18 251–270. 10.1177/1073858411403316 21677128 PMC3553600

[B3] Barthas F. Sellmeijer J. Hugel S. Waltisperger E. Barrot M. Yalcin I. (2015). The anterior cingulate cortex is a critical hub for pain-induced depression. *Biol. Psychiatry* 77 236–245. 10.1016/j.biopsych.2014.08.004 25433903

[B4] Bauer J. Werner A. Kohl W. Kugel H. Shushakova A. Pedersen A. (2018). Hyperactivity and impulsivity in adult attention-deficit/hyperactivity disorder is related to glutamatergic dysfunction in the anterior cingulate cortex. *World J. Biol. Psychiatry* 19 538–546. 10.1080/15622975.2016.1262060 27973969

[B5] Bousquet J. Schünemann H. J. Samolinski B. Demoly P. Baena-Cagnani C. E. Bachert C. (2012). Allergic rhinitis and its impact on asthma (ARIA): achievements in 10 years and future needs. *J. Allergy Clin. Immunol.* 130 1049–1062. 23040884 10.1016/j.jaci.2012.07.053

[B6] Brawley A. Silverman B. Kearney S. Guanzon D. Owens M. Bennett H. (2004). Allergic rhinitis in children with attention-deficit/hyperactivity disorder. *Ann. Allergy Asthma Immunol.* 92 663–667. 15237769 10.1016/S1081-1206(10)61434-2

[B7] Brożek J. L. Bousquet J. Agache I. Agarwal A. Bachert C. Bosnic-Anticevich S. (2017). Allergic rhinitis and its impact on asthma (ARIA) guidelines-2016 revision. *J. Allergy Clin. Immunol.* 140 950–958. 28602936 10.1016/j.jaci.2017.03.050

[B8] Callebaut I. Steelant B. Backaert W. Peeters R. Sunaert S. Van Oudenhove L. (2020). Brain activation after nasal histamine provocation in house dust mite allergic rhinitis patients. *Allergy* 76 1879–1882. 33283291 10.1111/all.14677 PMC8246755

[B9] Capuron L. Miller A. H. (2011). Immune system to brain signaling: neuropsychopharmacological implications. *Pharmacol. Ther.* 130 226–238. 10.1016/j.pharmthera.2011.01.014 21334376 PMC3072299

[B10] Capuron L. Pagnoni G. Demetrashvili M. Woolwine B. J. Nemeroff C. B. Berns G. S. (2005). Anterior cingulate activation and error processing during interferon-alpha treatment. *Biol. Psychiatry* 58 190–196. 10.1016/j.biopsych.2005.03.033 16084839 PMC1366492

[B11] Cavanna A. E. Trimble M. R. (2006). The precuneus: a review of its functional anatomy and behavioural correlates. *Brain* 129 564–583. 10.1093/brain/awl004 16399806

[B12] Chen K. Zheng X. Li Z. Xiang H. Chen B. Zhang H. (2019). Risk factors analysis of attention deficit/hyperactivity disorder and allergic rhinitis in children: a cross-sectional study. *Ital. J. Pediatr.* 45 99. 10.1186/s13052-019-0703-1 PMC6693261 31409392

[B13] Cheng L. Chen J. Fu Q. He S. Li H. Liu Z. (2018). Chinese Society of Allergy guidelines for diagnosis and treatment of allergic rhinitis. *Allergy Asthma Immunol. Res.* 10 300–353. 29949830 10.4168/aair.2018.10.4.300 PMC6021586

[B14] Cheng W. Rolls E. T. Qiu J. Liu W. Tang Y. Huang C. C. (2016). Medial reward and lateral non-reward orbitofrontal cortex circuits change in opposite directions in depression. *Brain* 139 3296–3309. 10.1093/brain/aww255 27742666

[B15] Cheng W. Rolls E. T. Ruan H. Feng J. (2018). Functional connectivities in the brain that mediate the association between depressive problems and sleep quality. *JAMA Psychiatry* 75 1052–1061. 10.1001/jamapsychiatry.2018.1941 30046833 PMC6233808

[B16] Cuffel B. Wamboldt M. Borish L. Kennedy S. Crystal-Peters J. (1999). Economic consequences of comorbid depression, anxiety, and allergic rhinitis. *Psychosomatics* 40 491–496. 10.1016/s0033-3182(99)71187-4 10581977

[B17] Cui Q. Sheng W. Chen Y. Pang Y. Lu F. Tang Q. (2020). Dynamic changes of amplitude of low-frequency fluctuations in patients with generalized anxiety disorder. *Hum. Brain Mapp.* 41 1667–1676. 10.1002/hbm.24902 31849148 PMC7267950

[B18] Cui Y. Jiao Y. Chen Y. C. Wang K. Gao B. Wen S. (2014). Altered spontaneous brain activity in type 2 diabetes: a resting-state functional MRI study. *Diabetes* 63 749–760. 10.2337/db13-0519 24353185

[B19] Damoiseaux J. S. Prater K. E. Miller B. L. Greicius M. D. (2012). Functional connectivity tracks clinical deterioration in Alzheimer’s disease. *Neurobiol. Aging* 33 .e819–.e830. 10.1016/j.neurobiolaging.2011.06.024 PMC3218226 21840627

[B20] De Virgilio A. Greco A. Fabbrini G. Inghilleri M. Rizzo M. I. Gallo A. (2016). Parkinson’s disease: autoimmunity and neuroinflammation. *Autoimmun. Rev.* 15 1005–1011. 27725149 10.1016/j.autrev.2016.09.027

[B21] Dodd J. W. Chung A. W. van den Broek M. D. Barrick T. R. Charlton R. A. Jones P. W. (2012). Brain structure and function in chronic obstructive pulmonary disease: a multimodal cranial magnetic resonance imaging study. *Am. J. Respir. Crit. Care Med.* 186 240–245. 10.1164/rccm.201202-0355oc 22652026

[B22] Etkin A. Egner T. Kalisch R. (2011). Emotional processing in anterior cingulate and medial prefrontal cortex. *Trends Cogn. Sci.* 15 85–93. 10.1016/j.tics.2010.11.004 21167765 PMC3035157

[B23] Eustache F. Piolino P. Giffard B. Viader F. De La Sayette V. Baron J. C. (2004). ‘In the course of time’: a PET study of the cerebral substrates of autobiographical amnesia in Alzheimer’s disease. *Brain* 127 1549–1560. 10.1093/brain/awh166 15102619

[B24] Fang B. J. Tonelli L. H. Soriano J. J. Postolache T. T. (2010). Disturbed sleep: linking allergic rhinitis, mood and suicidal behavior. *Front. Biosci. (Schol. Ed.)* 2:30–46. 10.2741/s44 20036927

[B25] Feng B. Jin H. Xiang H. Li B. Zheng X. Chen R. (2017). Association of pediatric allergic rhinitis with the ratings of attention-deficit/hyperactivity disorder. *Am. J. Rhinol. Allergy* 31 161–167. 10.2500/ajra.2017.31.4439 28490400

[B26] Fletcher P. C. Frith C. D. Baker S. C. Shallice T. Frackowiak R. S. Dolan R. J. (1995). The mind’s eye–precuneus activation in memory-related imagery. *Neuroimage* 2 195–200. 10.1006/nimg.1995.1025 9343602

[B27] Gelders G. Baekelandt V. Van der Perren A. (2018). Linking neuroinflammation and neurodegeneration in Parkinson’s disease. *J. Immunol. Res.* 2018 4784268. 10.1155/2018/4784268 PMC5926497 29850629

[B28] Gilmore A. W. Nelson S. M. McDermott K. B. (2015). A parietal memory network revealed by multiple MRI methods. *Trends Cogn. Sci.* 19 534–543. 10.1016/j.tics.2015.07.004 26254740

[B29] Godlewska B. R. Browning M. Norbury R. Igoumenou A. Cowen P. J. Harmer C. J. (2018). Predicting treatment response in depression: the role of anterior cingulate cortex. *Int. J. Neuropsychopharmacol.* 21 988–996. 10.1093/ijnp/pyy069 30124867 PMC6209854

[B30] Guo M. M. Wang L. J. Hsu T. Y. Yang K. D. Kuo H. C. (2020). Peanut sensitivity and allergic rhinitis in young children are associated with attention-deficit hyperactivity disorder symptoms in adolescence. *Neuropsychiatr. Dis. Treat.* 16 1349–1357. 10.2147/ndt.s232299 32547038 PMC7263365

[B31] Gur R. C. Turetsky B. I. Loughead J. Waxman J. Snyder W. Ragland J. D. (2007). Hemodynamic responses in neural circuitries for detection of visual target and novelty: an event-related fMRI study. *Hum. Brain Mapp.* 28 263–274. 10.1002/hbm.20319 17133387 PMC6871418

[B32] Hayden B. Y. Nair A. C. McCoy A. N. Platt M. L. (2008). Posterior cingulate cortex mediates outcome-contingent allocation of behavior. *Neuron* 60 19–25. 10.1016/j.neuron.2008.09.012 18940585 PMC2575690

[B33] Hebscher M. Ibrahim C. Gilboa A. (2020). Precuneus stimulation alters the neural dynamics of autobiographical memory retrieval. *Neuroimage* 210 116575. 10.1016/j.neuroimage.2020.116575 31972285

[B34] Hurwitz E. L. Morgenstern H. (1999). Cross-sectional associations of asthma, hay fever, and other allergies with major depression and low-back pain among adults aged 20-39 years in the United States. *Am. J. Epidemiol.* 150 1107–1116. 10.1093/oxfordjournals.aje.a009936 10568627

[B35] Kafkas A. Montaldi D. (2014). Two separate, but interacting, neural systems for familiarity and novelty detection: a dual-route mechanism. *Hippocampus* 24 516–527. 10.1002/hipo.22241 24436072

[B36] Kremer B. den Hartog H. M. Jolles J. (2002). Relationship between allergic rhinitis, disturbed cognitive functions and psychological well-being. *Clin. Exp. Allergy* 32 1310–1315. 10.1046/j.1365-2745.2002.01483.x 12220469

[B37] Lai C. H. (2018). The regional homogeneity of cingulate-precuneus regions: the putative biomarker for depression and anxiety. *J. Affect. Disord.* 229 171–176. 10.1016/j.jad.2017.12.086 29316519

[B38] Lee Y. H. Bak Y. Park C. H. Chung S. J. Yoo H. S. Baik K. (2020). Patterns of olfactory functional networks in Parkinson’s disease dementia and Alzheimer’s dementia. *Neurobiol. Aging* 89 63–70. 31980278 10.1016/j.neurobiolaging.2019.12.021

[B39] Léger D. Annesi-Maesano I. Carat F. Rugina M. Chanal I. Pribil C. (2006). Allergic rhinitis and its consequences on quality of sleep: an unexplored area. *Arch. Intern. Med.* 166 1744–1748. 10.1001/archinte.166.16.1744 16983053

[B40] Leibenluft E. (2017). Pediatric irritability: a systems neuroscience approach. *Trends Cogn. Sci.* 21 277–289. 10.1016/j.tics.2017.02.002 28274677 PMC5366079

[B41] Li R. Utevsky A. V. Huettel S. A. Braams B. R. Peters S. Crone E. A. (2019). Developmental maturation of the precuneus as a functional core of the default mode network. *J. Cogn. Neurosci.* 31 1506–1519. 10.1162/jocn_a_01426 31112473

[B42] Lundstrom B. N. Ingvar M. Petersson K. M. (2005). The role of precuneus and left inferior frontal cortex during source memory episodic retrieval. *Neuroimage* 27 824–834. 10.1016/j.neuroimage.2005.05.008 15982902

[B43] Lundstrom B. N. Petersson K. M. Andersson J. Johansson M. Fransson P. Ingvar M. (2003). Isolating the retrieval of imagined pictures during episodic memory: activation of the left precuneus and left prefrontal cortex. *Neuroimage* 20 1934–1943. 10.1016/j.neuroimage.2003.07.017 14683699

[B44] Lv H. Liu P. Zhou F. Gao Z. Fan W. Xu Y. (2021). TAK-242 ameliorates olfactory dysfunction in a mouse model of allergic rhinitis by inhibiting neuroinflammation in the olfactory bulb. *Int. Immunopharmacol.* 92:107368. 10.1016/j.intimp.2021.107368 33454639

[B45] Maddock R. J. Garrett A. S. Buonocore M. H. (2001). Remembering familiar people: the posterior cingulate cortex and autobiographical memory retrieval. *Neuroscience* 104 667–676. 10.1016/s0306-4522(01)00108-7 11440800

[B46] Maddock R. J. Garrett A. S. Buonocore M. H. (2003). Posterior cingulate cortex activation by emotional words: fMRI evidence from a valence decision task. *Hum. Brain Mapp.* 18 30–41. 10.1002/hbm.10075 12454910 PMC6871991

[B47] Marshall P. S. O’Hara C. Steinberg P. (2000). Effects of seasonal allergic rhinitis on selected cognitive abilities. *Ann. Allergy Asthma Immunol.* 84 403–410. 10.1016/s1081-1206(10)62273-9 10795648

[B48] Melamed I. Heffron M. (2016). Attention deficit disorder and allergic rhinitis: are they related? *J. Immunol. Res.* 2016 1596828. 10.1155/2016/1596828 PMC5107870 27872863

[B49] Meng Y. Wang C. Zhang L. (2019). Recent developments and highlights in allergic rhinitis. *Allergy* 74 2320–2328. 10.1111/all.14067 31571226

[B50] Miyazaki C. Koyama M. Ota E. Swa T. Mlunde L. B. Amiya R. M. (2017). Allergic diseases in children with attention deficit hyperactivity disorder: a systematic review and meta-analysis. *BMC Psychiatry* 17:120. 10.1186/s12888-017-1281-7 28359274 PMC5374627

[B51] Morita T. Tanabe H. C. Sasaki A. T. Shimada K. Kakigi R. Sadato N. (2014). The anterior insular and anterior cingulate cortices in emotional processing for self-face recognition. *Soc. Cogn. Affect. Neurosci.* 9 570–579. 10.1093/scan/nst011 23377900 PMC4014092

[B52] Naaijen J. Lythgoe D. J. Zwiers M. P. Hartman C. A. Hoekstra P. J. Buitelaar J. K. (2018). Anterior cingulate cortex glutamate and its association with striatal functioning during cognitive control. *Eur. Neuropsychopharmacol.* 28 381–391. 10.1016/j.euroneuro.2018.01.002 29395624

[B53] Ozdoganoglu T. Songu M. Inancli H. M. (2012). Quality of life in allergic rhinitis. *Ther. Adv. Respir. Dis.* 6 25–39. 22032987 10.1177/1753465811424425

[B54] Papapostolou G. Kiotseridis H. Romberg K. Dahl Å Lindgren M. (2020). Cognitive dysfunction and quality of life during pollen season in children with seasonal allergic rhinitis. *Pediatr. Allergy Immunol.* 32:67-76 10.1111/pai.13328 PMC7818136 32767782

[B55] Passamonti L. Tsvetanov K. A. Jones P. S. Bevan-Jones W. R. Arnold R. Borchert R. J. (2019). Neuroinflammation and functional connectivity in Alzheimer’s disease: interactive influences on cognitive performance. *J. Neurosci.* 39 7218–7226. 10.1523/jneurosci.2574-18.2019 31320450 PMC6733539

[B56] Raichle M. E. (2015). The brain’s default mode network. *Annu. Rev. Neurosci.* 38 433–447. 25938726 10.1146/annurev-neuro-071013-014030

[B57] Rhyou H. I. Nam Y. H. (2020). Association between cognitive function and asthma in adults. *Ann. Allergy Asthma Immunol.* 126 69–74. 10.1016/j.anai.2020.08.022 32858237

[B58] Robles-Figueroa M. Bedolla-Barajas M. Morales-Romero J. Pulido-Guillén N. A. Bustos-Gutiérrez L. R. M. (2020). Allergic rhinitis is associated with loss of energy and concentration difficulty: a cross-sectional study. *Am. J. Rhinol. Allergy* 34 108–114. 10.1177/1945892419877554 31558036

[B59] Rolls E. T. (2019). The cingulate cortex and limbic systems for emotion, action, and memory. *Brain Struct. Funct.* 224 3001–3018. 10.1007/s00429-019-01945-2 31451898 PMC6875144

[B60] Rolls E. T. Cheng W. Gong W. Qiu J. Zhou C. Zhang J. (2019). Functional connectivity of the anterior cingulate cortex in depression and in health. *Cereb. Cortex* 29 3617–3630. 10.1093/cercor/bhy236 30418547

[B61] Scarapicchia V. Garcia-Barrera M. MacDonald S. Gawryluk J. R. (2019). Resting state BOLD variability is linked to white matter vascular burden in healthy aging but not in older adults with subjective cognitive decline. *Front. Hum. Neurosci.* 13:429. 10.3389/fnhum.2019.00429 31920589 PMC6936515

[B62] Shenhav A. Botvinick M. M. Cohen J. D. (2013). The expected value of control: an integrative theory of anterior cingulate cortex function. *Neuron* 79 217–240. 10.1016/j.neuron.2013.07.007 23889930 PMC3767969

[B63] Sturm V. E. Sollberger M. Seeley W. W. Rankin K. P. Ascher E. A. Rosen H. J. (2013). Role of right pregenual anterior cingulate cortex in self-conscious emotional reactivity. *Soc. Cogn. Affect. Neurosci.* 8 468–474. 10.1093/scan/nss023 22345371 PMC3624960

[B64] Trikojat K. Luksch H. Rösen-Wolff A. Plessow F. Schmitt J. Buske-Kirschbaum A. (2017). “Allergic mood”–depressive and anxiety symptoms in patients with seasonal allergic rhinitis (SAR) and their association to inflammatory, endocrine, and allergic markers. *Brain Behav Immun* 65 202–209. 10.1016/j.bbi.2017.05.005 28495610

[B65] Vogt B. A. (2019). Cingulate impairments in ADHD: comorbidities, connections, and treatment. *Handb. Clin. Neurol.* 166 297–314. 10.1016/b978-0-444-64196-0.00016-9 31731917

[B66] Wamboldt M. Z. Hewitt J. K. Schmitz S. Wamboldt F. S. Räsänen M. Koskenvuo M. (2000). Familial association between allergic disorders and depression in adult Finnish twins. *Am. J. Med. Genet.* 96 146–153. 10.1002/(sici)1096-8628(20000403)96:2<146::aid-ajmg4>3.0.co;2-j 10893486

[B67] Wang L. J. Yu Y. H. Fu M. L. Yeh W. T. Hsu J. L. Yang Y. H. (2018). Attention deficit-hyperactivity disorder is associated with allergic symptoms and low levels of hemoglobin and serotonin. *Sci. Rep.* 8:10229. 10.1038/s41598-018-28702-5 PMC6035203 29980754

[B68] Yamasaki A. Burks C. A. Bhattacharyya N. (2020). Cognitive and quality of life-related burdens of illness in pediatric allergic airway disease. *Otolaryngol. Head Neck Surg.* 162 566–571. 10.1177/0194599820908202 32122241

[B69] Yang M. T. Chen C. C. Lee W. T. Liang J. S. Fu W. M. Yang Y. H. (2016). Attention-deficit/hyperactivity disorder-related symptoms improved with allergic rhinitis treatment in children. *Am. J. Rhinol. Allergy* 30 209–214. 10.2500/ajra.2016.30.4301 27216352

[B70] Yang S. Wu J. Zhang Q. Li X. Liu D. Zeng B. (2018). Allergic rhinitis in rats is associated with an inflammatory response of the hippocampus. *Behav. Neurol.* 2018:8750464. 10.1155/2018/8750464 PMC5926495 29849816

[B71] Zhang T. Yu G. D. Gu P. Tang Q. Jin Y. He X. C. (2020). [Evaluation and analysis of anxiety, depression and quality of life in vasomotor rhinitis]. *Zhonghua Er. Bi Yan. Hou. Tou. Jing. Wai. Ke Za Zhi.* 55 769–773. 32791776 10.3760/cma.j.cn115330-20191230-00787

